# Preprint citation practice in PLOS

**DOI:** 10.1007/s11192-022-04388-5

**Published:** 2022-05-25

**Authors:** Marc Bertin, Iana Atanassova

**Affiliations:** 1 ELICO Laboratory, Université Claude Bernard Lyon 1, Bâtiment Nautibus - 43 Boulevard du 11 novembre 1918, 69622 Villeurbanne cedex, France; 2 CRIT Laboratory, Université de Bourgogne Franche-Comté, 30 rue Mégevand, 25000 Besançon, France; 3 Institut Universitaire de France (IUF), Paris, France

**Keywords:** Preprint, Citation contexts, Correspondence analysis, PLOS, IMRaD

## Abstract

The role of preprints in the scientific production and their part in citations have been growing over the past 10 years. In this paper we study preprint citations in several different aspects: the progression of preprint citations over time, their relative frequencies in relation to the IMRaD structure of articles, their distributions over time, per preprint database and per PLOS journal. We have processed the PLOS corpus that covers 7 journals and a total of about 240,000 articles up to January 2021, and produced a dataset of 8460 preprint citation contexts that cite 12 different preprint databases. Our results show that preprint citations are found with the highest frequency in the Method section of articles, though small variations exist with respect to journals. The PLOS Computational Biology journal stands out as it contains more than three times more preprint citations than any other PLOS journal. The relative parts of the different preprint databases are also examined. While ArXiv and bioRxiv are the most frequent citation sources, bioRxiv’s disciplinary nature can be observed as it is the source of more than 70% of preprint citations in PLOS Biology, PLOS Genetics and PLOS Pathogens. We have also compared the lexical content of preprint citation contexts to the citation content to peer-reviewed publications. Finally, by performing a lexicometric analysis, we have shown that preprint citation contexts differ significantly from citation contexts of peer-reviewed publications. This confirms that authors make use of different lexical content when citing preprints compared to the rest of citations.

## Introduction

In recent years, the growing role of preprints in the publication process and the creation of a large number of publicly available preprint databases led to an increasing interest in the research community in scientometrics [see Berg (2017), da Silva (2017a, 2017b, 2017c), Kaiser (2017)]. Indeed, a growing number of online repositories provide access to new research that is still in production and not yet validated by peers. This contributes to the acceleration of the dissemination of scientific results (Abdill and Blekhman 2019; Larivière et al. 2014). The arrival of bioRxiv following the ArXiv model and its operation [see the work of Pinfield (2001) on the use of this type of service by physicists], is presented by Berg et al. (2016). Also, Fu and Hughey (2019) show that having published a paper in bioArxiv is correlated with a high altmetric value.

The debate around preprints is important due to their complex nature. Preprint databases such as bioRxiv were initially intended to allow authors to receive feedback from peers and thus to improve their papers before their submission to peer reviewed journals (Anderson, 2020). However, citing unverified work or nonvalidated facts may prove problematic as highlighted by the work of da Silva (2018). Finally, a significant proportion of the preprints are never published in peer-reviewed journals (Abdill & Blekhman, 2019).

In times of crisis when research results need to be disseminated rapidly, preprints play an important role (Añazco et al., 2021). As health crises mobilize research efforts, the role of preprints as an underutilized mechanism for accelerating the dissemination of scientific findings is discussed by Johansson et al. (2018) and Majumder and Mandl (2020). More broadly, this phenomenon has invited community researchers to produce datasets to include the production of arXiv in a recommendation system (Saier & Färber, 2019).

According to the work of Fraser et al. (2021), COVID-19 preprints are consulted and distributed at least 15 times more than preprints that do not deal with COVID-19. This redefines the role and place of preprints in the world of scientific publishing and invites a reflection on the practices. Preprints have an informative role, and in times of crisis, practices can change, as shown by Bordignon et al. (2021) who propose a comparative study around the response to the COVID-19 pandemic. Their study shows that the percentage of structured abstracts has decreased in favour of non-structured abstracts during the pandemic for some preprint servers. In times of pandemic, the ease of use, review practices, and acceptance policies of preprinted manuscripts vary (Nabavi Nouri et al., 2021). The authors of this article note that, if preprints are part of the future of science, users will need to appreciate not only their usefulness, but also their limitations.

Preprints have become an important source of information for COVID-19 stakeholders, including traditional media, social media, and policymakers. The work of Ravinetto et al. (2021) focus on the ethical dimension of this issue. They point out that, despite warnings about the nature of preprints, many users may still confuse them with peer-reviewed manuscripts. If unconfirmed but already widely reported results from a preprint subsequently turn out to be wrong or misinterpreted, it can be very difficult to “unlearn” what one believed. For this reason, the authors propose recommendations for good practices.

On the level of a country, a study of Korea shows the penetration rate of preprints (Jung & Sun, 2021), and report that, while preprints are not very common, more than half of the respondents of the surveys have favorable attitudes towards preprints. This may lead to a consensus to make preprint policies acceptable to publishers in Korea.

Traditionally, the knowledge built through scientific articles relies on originality and refers to previous and peer-reviewed work, thus participating in the cumulative structure of scientific knowledge through the act of citations. In fact, Fry et al. (2019) show that the purposes of preprints are diverse: from increased dissemination speeds for authors to an invitation to critical reading for work that has not been peer-reviewed. The question that arises today is the place of preprints alongside scientific articles and their citations: how do preprints contribute to the construction of new scientific knowledge while their validity, as sources, is not established by peer review? Will the use of preprint citations reinforce the critical attitude expressed in articles or will it weaken the edifice of knowledge? To answer these questions, one of the necessary steps is to study the nature and place of preprints in scientific journals.

Preprints are frequently present in peer-reviewed publications as cited references. Direct citations to preprint databases such as arXiv, RePEc, SSRN and PMC all increased steadily from 2000 to 2013 according to the Scopus study by Li et al. (2015). Also, Fraser et al. (2020) show that papers submitted to bioRxiv receive more attention from the scientific community than other publications. This implies that in the publication process, the citation of works from preprint databases is becoming now a part of the publication cycle (Hoy, 2020; Penfold & Polka, 2020). For this reason, Desjardins-Proulx et al. (2013) question the relationship between the preprint databases and the publishers with regard to the quality, but also the relevance of this type of production. Other ethical considerations arise such as plagiarism (Giles, 2003). While citations to preprints play an essential role in research, the links between preprints and existing peer-reviewed publications are lacking. Recently, the work of Cabanac et al. (2021) contributes to establishing preprint-publication links. Such studies can provide information to allow a better interpretation of the citation contexts of preprints.

In this paper, we propose to study the citations of preprints, and to compare them to citations of peer-reviewed publications. More precisely, we focus on the frequency and relative part of preprint citations in peer-reviewed articles, as well as their evolution over time. Preprints contain recent methods and results that have not yet been peer-reviewed. For this reason, the knowledge on how such methods and results are actually cited and used in peer-reviewed journals will provide an important component of understanding the construction of new knowledge.

We use as dataset the 7 PLOS journals, that are mainly in the biomedical field. We analyse both types of citations according to their position in the different journals and in the Introduction, Method, Results and Discussion (IMRaD) structure of articles. Our study aims to characterize preprint citations in terms of their frequency over the last 10 years, their positions in the rhetorical structure of articles, and their relative number compared to the rest of citations. Furthermore, studying the positions of preprint citations in the IMRaD structure of articles, allows us to understand the ways in which they are leveraged by researchers and the place that preprints hold in scientific argumentation. Finally, from a qualitative perspective, we examine citation contexts of preprint citations and compare them to citation contexts of peer-reviewed publications using a lexicometric approach. This analysis allows us to find out whether the use of preprint citations differs, in terms of lexical content, from traditional citations to peer-reviewed publications.

The rest of the article is organised as follows: the next section presents the methods that were used to construct the dataset of preprint citation contexts and to perform the lexicometric analysis of the data. “Results” section presents the main results of this study. We report on the progression of citations over time, their frequency in the four sections of IMRaD and relative part related to all citations. Then we study the contributions of the different preprint databases in the PLOS journals. The final two subsections, “Verbs in preprint citation contexts” and “Correspondence analysis of citation contexts”, are dedicated to the qualitative study of preprint citation contexts: the verbs that appear in preprint citation contexts, and a lexicometric analysis of preprint citation contexts compared to citations to peer-reviewed publications. “Discussion” section discusses the outcomes of this study and the last seciton provides a conclusion.

## Methods

### Creation of the dataset of preprint citation contexts

We have processed all research articles published by PLOS up to January 2021. This corpus contains seven different journals, and the majority of the articles is in the biomedical domain. The largest journal, PLOS ONE, is multidisciplinary and contains also articles in various other domains. The PLOS corpus is available in XML format following the JATS (Journal Article Tag Set) XML schema, that is specifically designed for the representation and processing of scientific papers.

Table 1 presents the number of articles and sentences per journal and the total size of the PLOS corpus.

**Table 1:** PLOS corpus (up to January 2021)

Journal	Nb of articles	Nb of sentences
PLOS ONE	214,732	41,084,599
PLOS Genetics	7211	1,888,689
PLOS Neglected Tropical Diseases	6202	1,188,239
PLOS Pathogens	6213	1,661,449
PLOS Computational Biology	5860	1,688,426
PLOS Biology	2828	751,489
PLOS Medicine	1742	327,025
Total	244,788	48,589,916

The editorial requirements of PLOS journals impose using the IMRaD structure for research articles. As a consequence, the vast majority of the articles follow this pattern. While the four main section types (Introduction, Method, Results, Discussion) are present in almost all articles, the order of these sections can vary according to the discipline and the journal. In our experiment, we have considered only research articles that follow this IMRaD pattern.

Our processing pipeline contains the following main steps:

1. Extraction of article metadata and bibliography items;
2. Section classification and processing of section content: paragraph and sentence segmentation;
3. Processing of citations and linking them to bibliography items;
4. Tagging citations as preprint and non-preprint citations;
5. Extraction of sentences containing citations and their positions in the sections;
6. Correspondence Analyses (CA) of preprint and non-preprint citation contexts.

#### Section classification

We classify the sections into the four main section types (”Introduction”, ”Method”, ”Results” and ”Discussion”). About 57% of the sections in the dataset are labelled as ”intro”, ”method”, ”results” etc. in the ”sec-type” attribute of the XML section element. We used these labels where possible. The remaining sections were classified using their section titles. To do this, sets of regular expressions were manually designed to account for the possible variations in the titles. For example, the Discussion section was found under 488 different titles such as ‘Discussions’, ‘Discussion on practical implementation of proposed handover strategy’, ‘Summary and Discussion’, ‘Discussion and implications’, ‘General Discussion’, ‘Strengths and Limitations of the Study’, ‘Discussion/Conclusion’, etc.

Some of the articles contain sections with titles that are subject specific and could not be classified in this way. Such articles were left out. Among the total of 263,542 articles in the PLOS corpus, 234,592 (89%) articles contain the four section types and were used for our study.

#### Processing and identification of preprint citations

Article content was segmented into paragraphs and sentences. Citations were identified, their metadata were extracted and linked to bibliography items using the existing annotations in the XML source files (i.e. xref elements that point to bibliography items).

The presence of citation ranges in the texts (e.g. [7]–[11]) requires supplementary processing. In fact, each such range in represented in the XML structure of the document by only two xref elements that are linked to bibliography items. Citations with numbers inside the range (e.g. [8], [9] and [10] in the range [7]–[11]) do not have xref elements in the XML. After the creation of these new links, we found out that they account for about 9.5% of all citations in the dataset. Table 2 presents the number of bibliography items and citations per journal in our dataset.

**Table 2:** PLOS dataset: IMRaD articles, bibliography items and citations per journal

Journal	Articles (IMRaD)	Bibligraphy items	Citations
PLOS ONE	204,981	9,849,669	13,682,522
PLOS Genetics	7173	453,315	693,201
PLOS Neglected Tropical Diseases	6168	299,917	422,065
PLOS Pathogens	6201	391,411	592,478
PLOS Computational Biology	5502	327,410	533,204
PLOS Biology	2796	175,833	281,448
PLOS Medicine	1728	86,686	133,719
Total	234,549	11,584,241	16,338,637

To identify preprint citations, first we compiled a list of the names of known preprint databases. In order to account for all preprint databases, we used the lists of repositories from several sources^1^. Preprint citations were then identified by processing their bibliography items’ metadata and matching them against regular expressions designed to cover the list of preprint databases.

Preprint databases can be of various types: multidisciplinary, thematic, linked to a publisher, etc. We account for this diversity in Table 3 that lists the preprint databases and the number of citations found in the dataset for each database. From the entire list of preprint databases, we identified 12 different sources that are cited in our dataset.

**Table 3:** Preprint databases

Preprint database	Discipline(s)	Created	Nb of citations
arXiv	Multidisciplinary	1991	4759
SSRN		1994	575
CogPrints		1997–2017	9
Nature Precedings		2007–2012	114
ViXra		2009	22
AAS Open Research		2018	2
Preprint*			254
Cryptology ePrint Archive	Cryptography	1996	11
RePEc	Economics	1997	198
PeerJ preprint	Biology, medicine	2013–2019	137
bioRxiv	Biology	2013	2363
PsyArXiv	Psychology	2016	15
SocArXiv	Social science	2016	1
			Total: 8460

*The ‘preprint’ keyword appearing in the bibliography item source without the mention of a specific database

#### Dataset

Following the steps described above, we have produced our dataset by extracting all 8460 preprint citations of the PLOS corpus, together with their citation contexts and positions within the IMRaD structure of papers. This dataset is available in Open Access on Zenodo (Atanassova & Bertin, 2022).

### Analysis of citation contexts

One important research problem is the comparison between citation contexts containing references to preprints and the rest of citation contexts, in terms of their linguistic characteristics. To compare these two classes of citation contexts, we have chosen to carry out a lexicometric experiment (Benzécri, 1992, 1973), called Correspondence Analysis, that can indicate whether the lexical content of the two classes differs in terms of the distributions of the forms.

Correspondence Analysis (CA) is a geometric approach to visualize the rows and columns of a contingency table in a scatter plot, so that the positions of the row points and the column points correspond to their associations in the table. The contingency table contains the frequencies formed by the two variables. These coordinates therefore allow the association between the row and column items to be visualized graphically in a two-dimensional graph. What interests us in this type of approach is that the CA is based on calculations of the inertia of the word cloud that constitutes a corpus. The goal of CA is to represent a maximum of the total inertia on the first factorial axis, a maximum of the residual inertia on the second axis, and so on until the last dimension. This has the consequence of displaying graphically oppositions and similarities.

Within the context of this study, we consider as citation contexts the sentences that contain the citations. All citation contexts in the collections were split into words and lemmatised: all different forms of a lexical item are identified and associated with the same lexical item. From a technical point of view, we used the IRaMuTeQ software (Ratinaud & Déjean, 2009) to perform dictionary-based processing, without disambiguation, also called endogenous lemmatisation. After lemmatisation, we filtered out only the verbal, nominal and adjectival forms. The CA visualizations were produced using the R FactoMineR package (Lê et al., 2008).

As our dataset contains 8460 preprint citation contexts, to compare them with non-preprint citation contexts we need to consider collections of similar sizes. From the entire set of more than 16M citations to peer-reviewed publications in PLOS, we constructed 3 different collections (c1, c2 and c3) of 8460 citations to peer-reviewed publications chosen randomly. The linguistic difference of citing sentences between preprint and peer-reviewed papers may be biased by journals. For this reason, the three random collections have been designed so that the citations to peer-reviewed publications are consistent with the proportion of preprint citations among the different journals. Thus, the four collections have the same distribution of citations across journals.

Thus we obtain four collections of citations contexts having the same size. One collection contains only preprint citation contexts, and the other three contain citations to peer-reviewed publications. These four collections were used to perform the CA and visualise the differences and similarities between the collections.

## Results

### Progression of citations over time

**Fig. 1:**
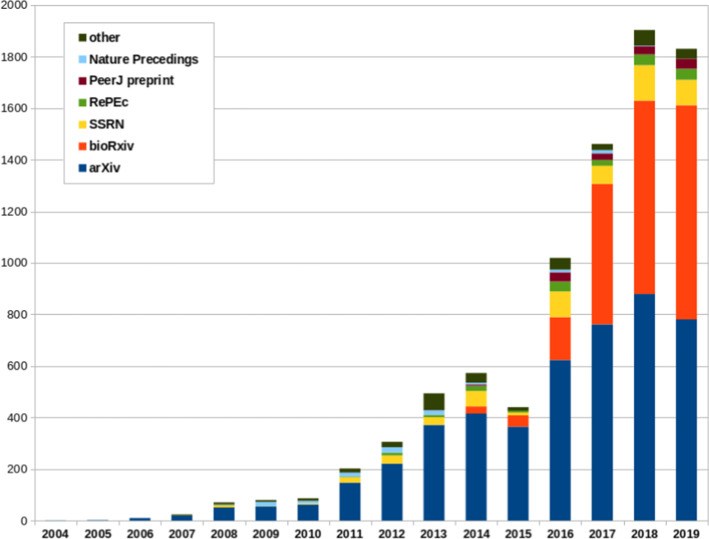
Progression of the number of preprint citations by year

We have plotted the number of preprint citations received by the different preprint databases by year. Figure 1 shows that the overall number of preprint citations grows rapidly since 2011 and this growth is consistent with the creation and development of new preprint repositories. For example, the bioRxiv database was created in 2013 and its presence in the citations is visible from 2014 and has been growing ever since.

**Fig. 2:**
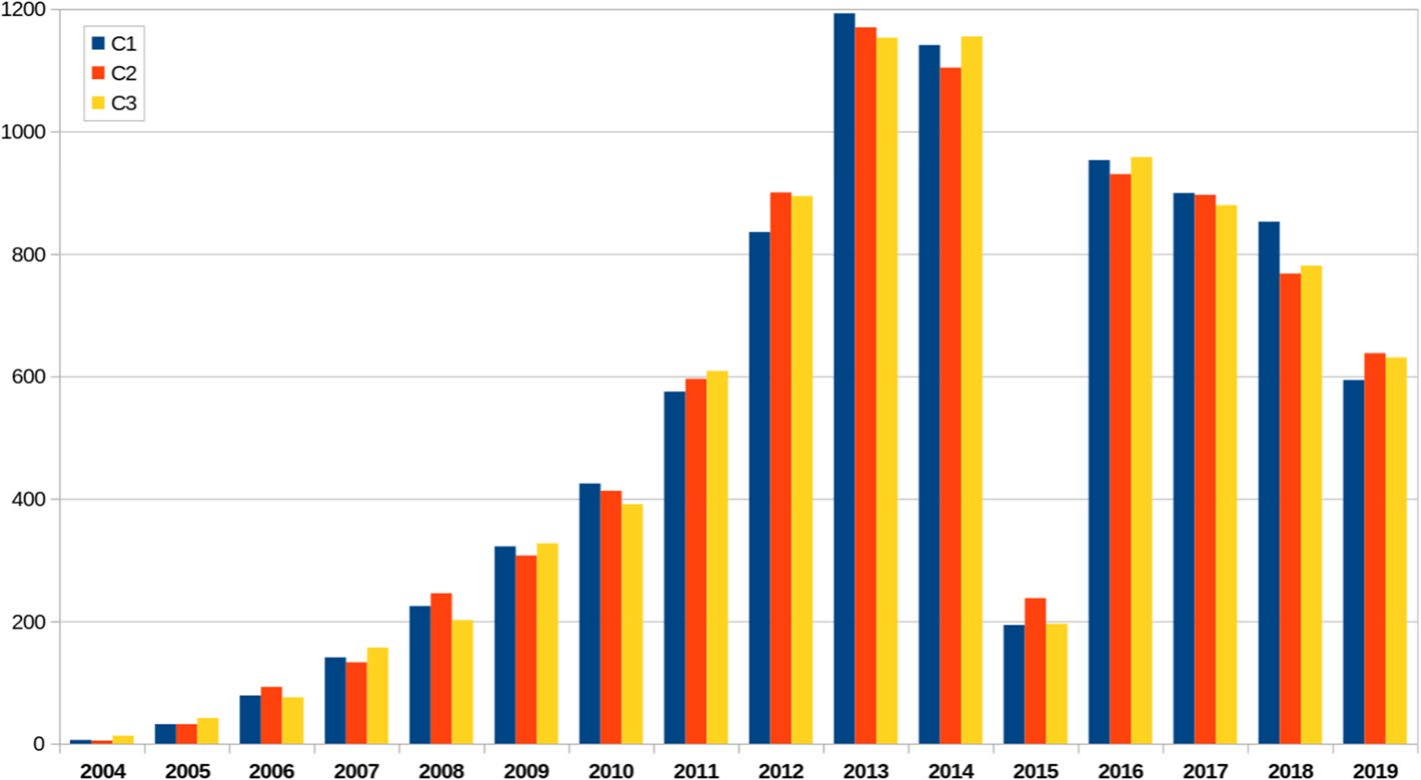
Progression of the number of citations by year: collections c1, c2 and c3 of citations to peer-reviewed publications

In order to compare the progression of preprint citation over time with that of the rest of citations, we have considered three different random collections of citation contexts of peer-reviewed publications (c1, c2 and c3). “Analysis of citation contexts” section provides details on the construction of collections c1, c2 and c3. Figure 2 shows the progression of citations over time in these three collections. We observe that the two progressions are quite different, especially after 2013. In fact, the relative part of peer-reviewed citations is the highest in 2013 and 2014, while for pre-print citations it is highest in 2018 and 2019.

### Frequency of preprint citations in the sections of the IMRaD structure

**Fig. 3:**
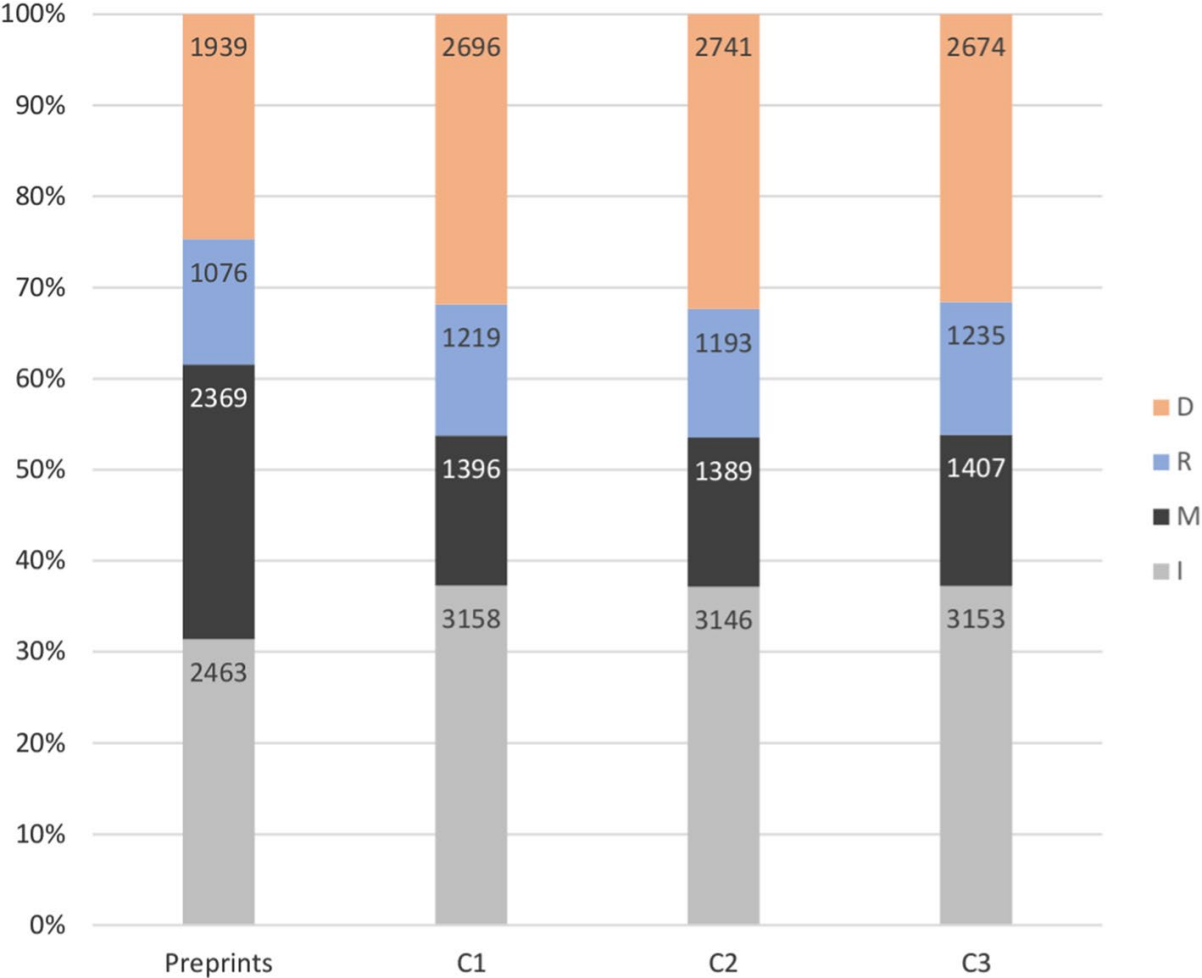
Relative part of preprint citations in the four sections of the IMRaD structure, compared to the three collections of citations to peer-reviewed publications

We have examined the positions of preprint citations with respect to the IMRaD structure and in the different PLOS journals. Figure 3 presents the relative part of preprint citations in the four sections of the IMRaD structure, and also the relative parts of citations of the three collections c1, c2 and c3 in the four sections. This figure shows that the preprint citations tend to appear more often in the Methods section compared to the rest of cittaions. This means that the number of preprint citations in the Methods section is much higher that what we can expect for the rest of citations.

**Fig. 4:**
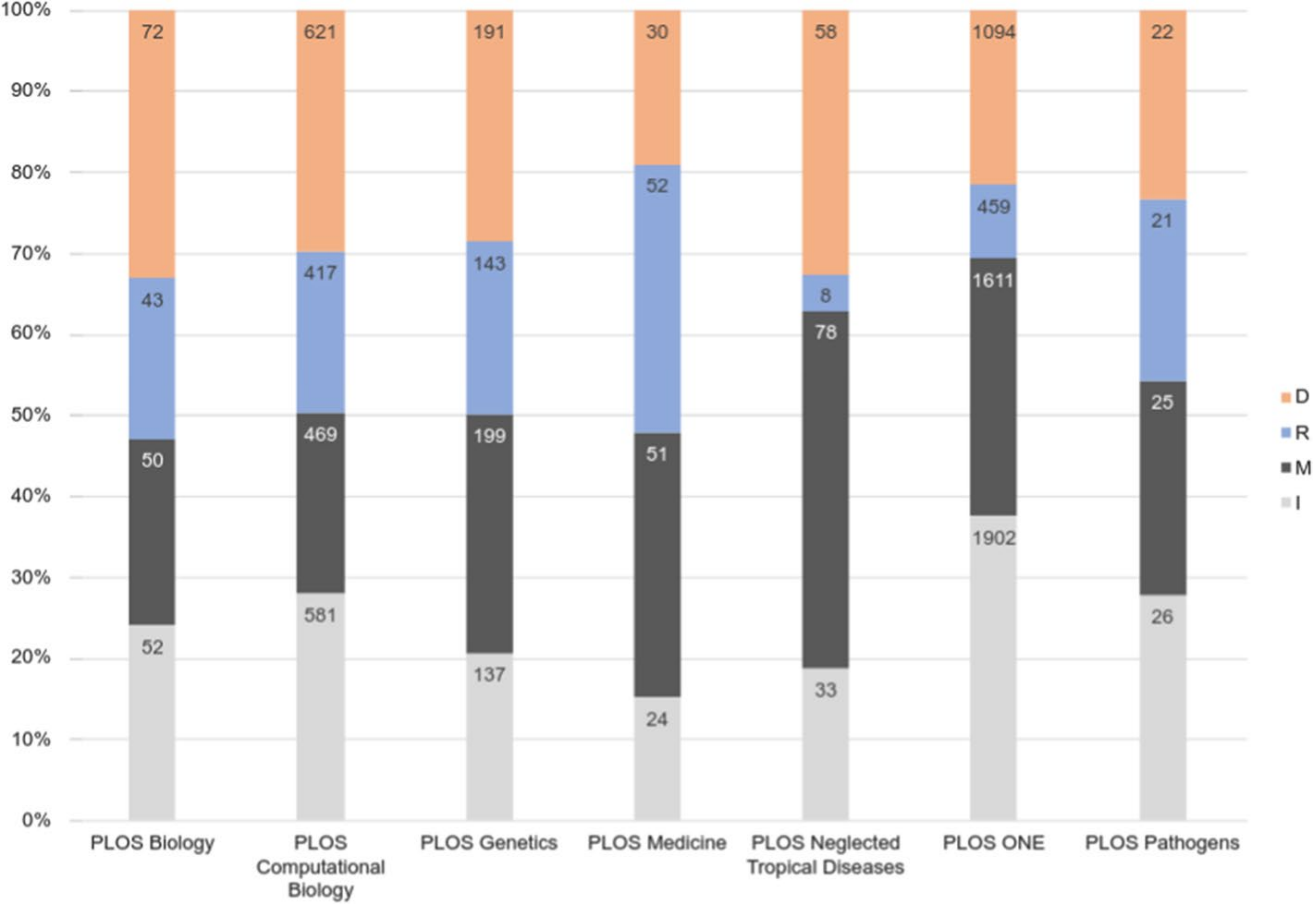
Relative part of preprint citations in the four sections of the IMRaD structure for each journal

**Fig. 5:**
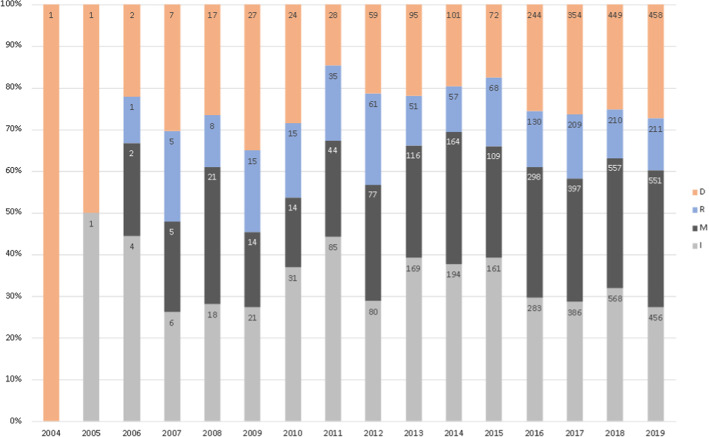
Evolution over time of the relative part of preprint citations in the IMRaD structure

Figure 4 presents the relative parts of preprint citations in the different sections for each journal. Figure 5 shows the evolution of the places of preprint citations in the IMRaD structure over time.

The first important observation is the part of preprint citations in the Method section. In fact, other studies on the distribution of citations in the IMRaD structure (Bertin et al., 2016) have shown that the Method section contains the smallest number of citations in the articles, the biggest number of citations being, naturally, in the Introduction. The fact that between 22 and 44% of preprint citations are found in the Method section is particularly interesting and means that preprint citations have distributions that are very different from those of the rest of the citations. Furthermore, on Fig. 5 we can observe that citations to preprints in the Method section have been growing steadily since 2005.

The Result section contains between 10 and 20% of all preprint citations since 2006. However, we note a relatively large number of preprint citations in the Result section in one particular journal, PLOS Medicine. This suggests strong disciplinary differences when citing preprints. In fact, an established practice in Medicine might be to cite the results of similar on-going experiments or to refer to the preprint version of the published paper.

The Introduction section contains the largest part of preprint citations for the PLOS One journal, unlike the rest of the journals. As PLOS One contains the largest number of articles and citations in the dataset, the data on Fig. 5 reflects mainly the evolution of preprint citations in this journal. We observe that between 2005 and 2010, the Introduction and Discussion sections share almost equal parts of preprint citations. From 2011 to 2015, the Introduction section contains about twice more preprint citations than the Discussion section. And since 2016, the relative parts of preprint citations in the Introduction and Discussion are again almost equal.

**Fig. 6:**
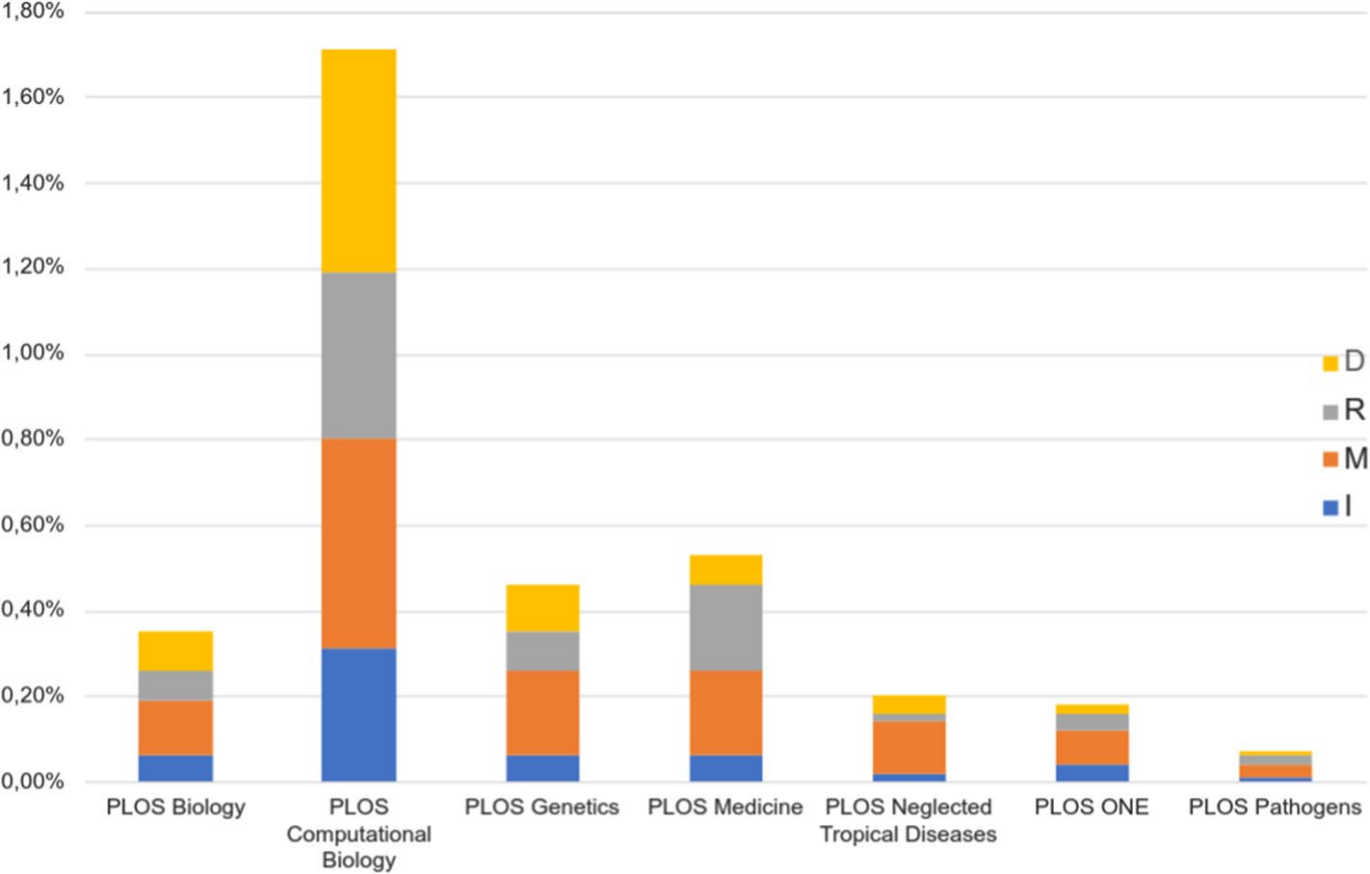
Preprint citations as a percentage of all citations in IMRaD

To complete these results, Fig. 6 shows the relative part of preprint citations as a percentage of all citations in each section and journal. Preprint citations are particularly frequent in the Method section, and somewhat less frequent in the Result and Discussion sections, except for PLOS Computational Biology. The journal PLOS Computational Biology stands out on this graph as having a very high relative number of preprint citations. Also, we note that in this journal the parts of citations in the Method and Discussion sections are almost the same, unlike the rest of the journals.

### Contribution of the different preprint databases

**Fig. 7:**
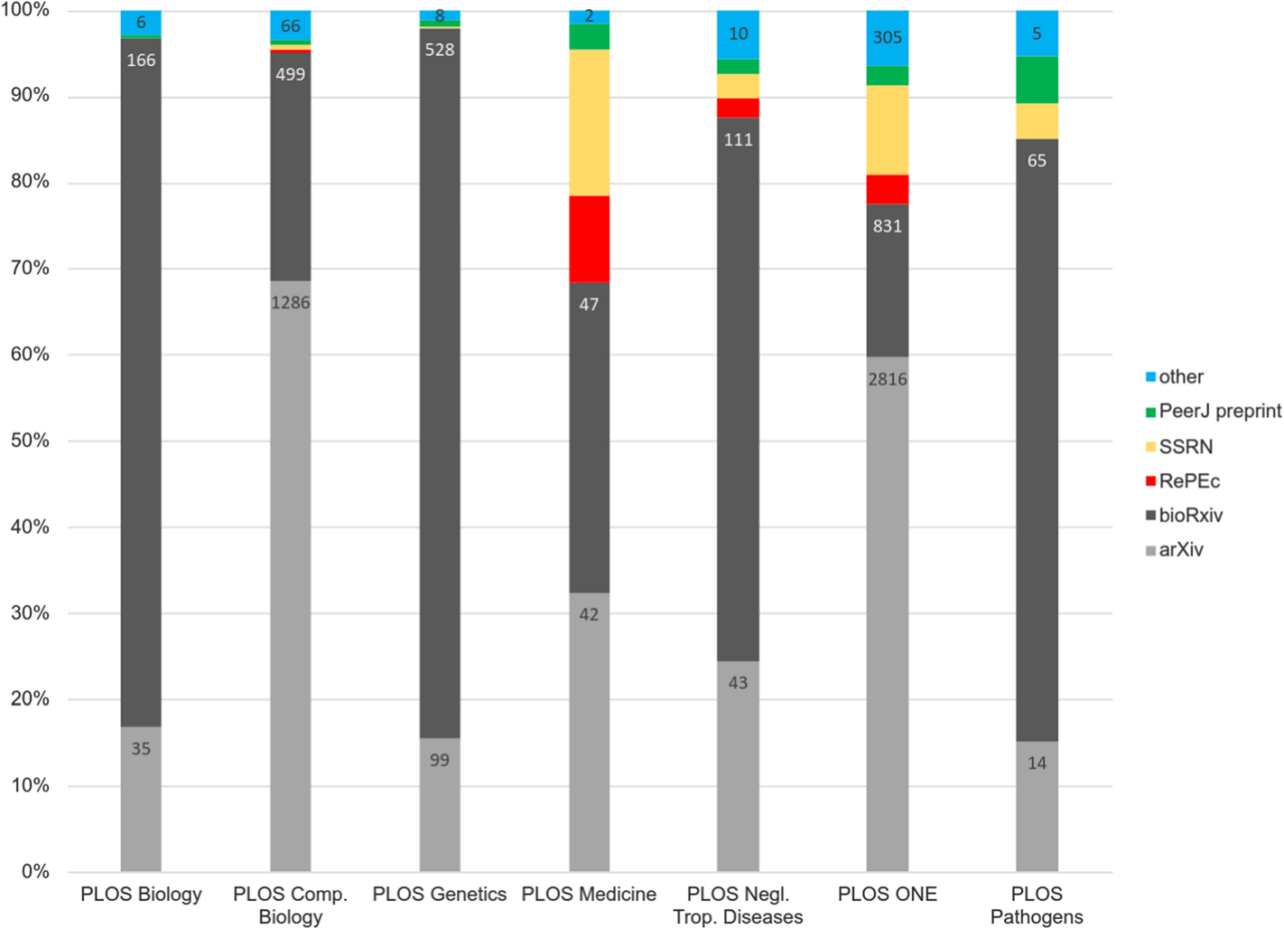
Contribution of preprint databases to preprint citations

The disciplinary nature of the different preprint databases is one of the factors that result in the different number of preprint citations among the journals. Figure 7 presents the relative parts of the contribution of the different preprint databases to the total preprint citations in the PLOS journals. We can observe that BioRxiv is present in all PLOS journals, and is most frequently cited in PLOS Biology, PLOS Genetics, PLOS Neglected Tropical Diseases and PLOS Pathogens. Thus, the disciplinary speciality in the biomedical field leads to the wide use of this preprint database by researchers. These data corroborate the results obtained by Li et al. (2015) who identified the point at which repositories are cited in their own domain.

### Verbs in preprint citation contexts

A first analysis of the verbs that appear in citations contexts in PLOS journals shows the singular nature of the Methods section. Considering all citation contexts, a study by Bertin and Atanassova (2014) shows that the most frequent verbs in the Methods section differ significantly from those used in the other sections of articles. The top 20 verbs for the Methods section are: *use, describe, perform, follow, obtain, generate, base, determine, contain, calculate, carry, identify, accord, include, express, estimate, see, measure, analyse, prepare*.

In a similar perspective, we have studied the verb frequencies in the citation contexts of preprints, i.e. in citing sentences. As a result, we observe that the 20 most frequent verbs in the Methods section are: ***use***, ***perform***, *study*, ***describe***, ***calculate***, ***obtain***, ***include***, ***analyse***, *develop*, *report*, *select*, ***determine***, *order*, *scale*, *mix*, *consider*, *make*, *represent*, *reduce*, *research*. The 8 verbs that are common with the previous list are presented in bold. We note in particular that the verb *study*, which is the third most common in preprint citation contexts, does not belong to the list of the 20 most frequent verbs in all citation contexts. Verbs such as *study, develop, report* are present in preprint citation contexts, while *follow* and *generate* are not.

The fact that the list of verbs used in preprint citation contexts is very different from the one observed in all citation contexts, indicates that preprint citation contexts have linguistic properties that distinguish them from the rest of citation contexts in terms of lexical content. For this reason, we can hypothesize that preprint citation contexts make use of specific linguistic patterns. To further characterise this phenomenon, we need to compare their full lexical content to citation contexts of peer-reviewed publications, and we do this in the following Sect. 3.5.

### Correspondence analysis of citation contexts

We perform Correspondence Analysis (CA) on four collections of citation contexts having the same size: the collection of preprint citation contexts and three different random collections of citation contexts of peer-reviewed publications (c1, c2 and c3, see Sect. 2.2). Using CA, we produce two different visualizations, one comparing the different IMRaD sections for all collections, and one comparing the journals for all collections.

Correspondence analysis provides a synthetic view of the “salient” information contained in a contingency table. The center of the projected cloud corresponds to the average profile. Thus, a position far from the center means that the particular object is differs greatly from the average profile.

**Fig. 8:**
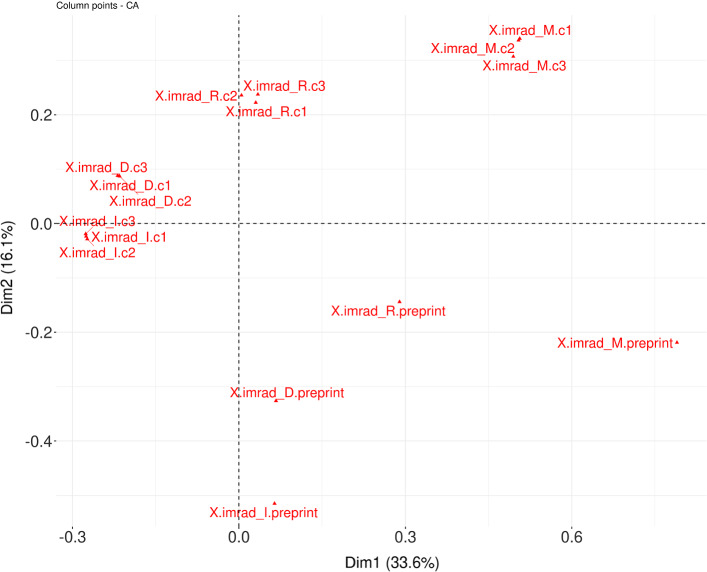
CA applied to preprint citation contexts with citation contexts of peer-reviewed publications in IMRaD

The Fig. 8 shows a CA of the four collections in relation to the sections in the IMRaD structure. The proximity between the items labelled c1, c2 and c3 means that the citation contexts that appear in the sections in these three collections are somewhat similar in terms of lexical content. In the case of the distributions of IMRaD structure versus the other three collections, we observe that the IMRaD structures of the preprints are far away from the collections c1, c2 and c3. Furthermore, preprint citation contexts are plotted in the lower right part of this graph. They are vertically separated from the rest of the citation contexts and isolated in the same quadrant, meaning that their lexical content is significantly different from that of the other citation contexts.

**Fig. 9:**
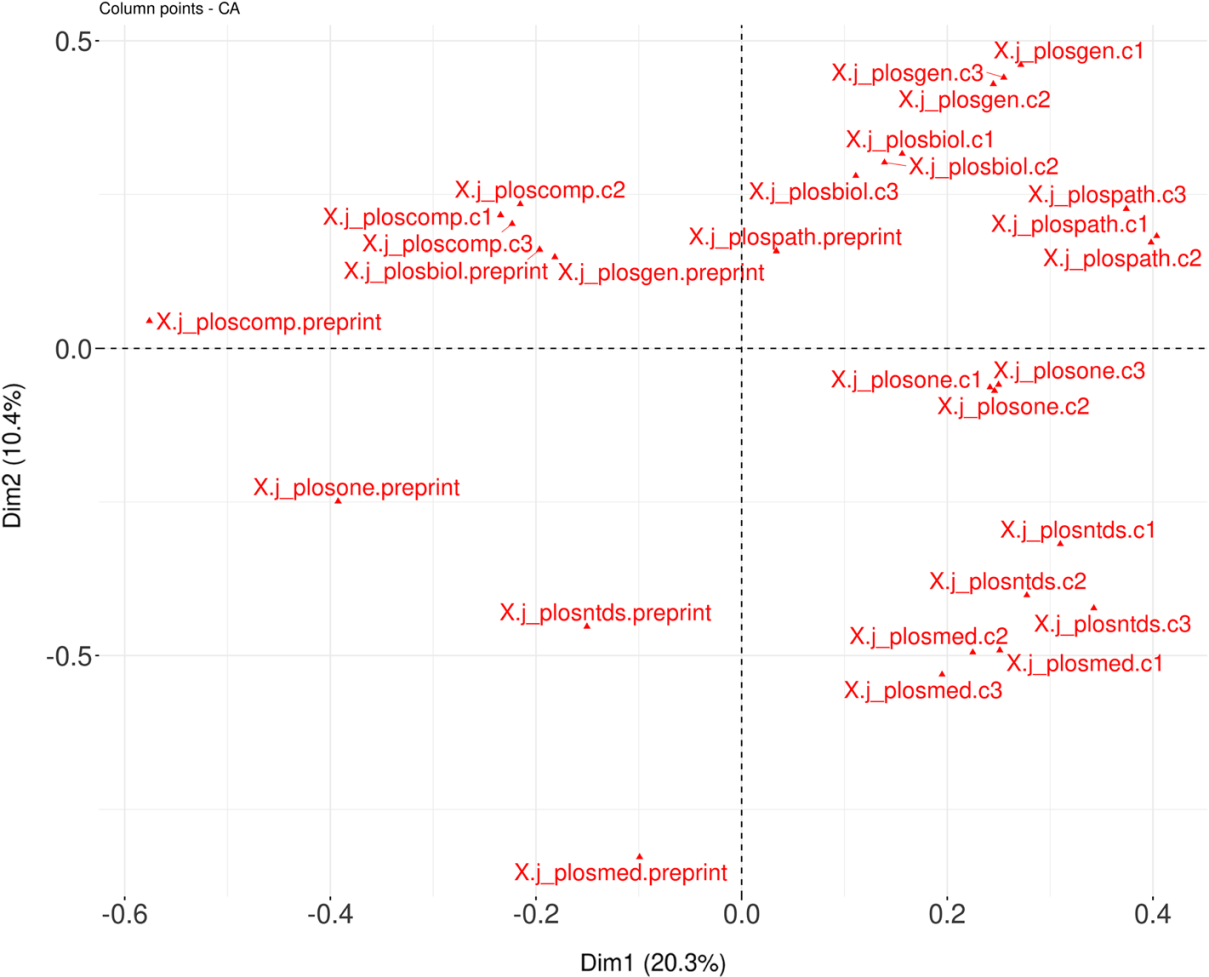
CA applied to preprint citation contexts with citation contexts of peer-reviewed publications in the PLOS Journals

The Fig. 9 shows a CA of the four collections according to the different journals in which they appear. We can observe, again, that all elements of the three collections c1, c2 and c3 are plotted closely together. Preprint citation contexts are in the left part of this graph, relatively to the left of the rest of citation contexts. Preprint citation contexts appear on this graph relatively far away (shifted to the left and down) from the corresponding citation contexts from the c1, c2 and c3 collections.

These results show how atypical preprint citations are, in terms of both their distributions in the IMRaD structure and the different journals, and most importantly, the lexical content of the citation contexts. The specificities that we have discovered invite a wider reflection on the nature of preprints in a citation context.

## Discussion

We have shown that preprint citations gain more and more importance over the last years and this means that preprints have been actively used by the community. Naturally, the growth in the number of preprint citations over time is consistent with the creation and growth of preprint repositories.

Preprints constitute a new citation object that needs to be taken into consideration in future studies as they represent a new paradigmatic shift. Their particularity stems from the following two reasons:

1. a preprint might or might not have the quality to be published as peer-reviewed article, or might simply not be intended for this purpose;
2. a preprint citation must include reference to the version of the preprint. Indeed, a preprint can be modified over time, with no restrictions to the depth and significance of such modifications.

The position of preprint citations in the IMRaD structure shows that these citations do not follow the same distribution as the rest of the citations in the articles. Such distributions for citations to peer-reviewed papers have already been established in the literature around PLOS (Bertin et al., 2016). Considering preprint citations, an intuitive hypothesis could be that non-validated knowledge is placed in the introduction or background of an article. Also, we could expect to find preprint citations, naturally, in the discussion section where authors confront their results with other ongoing experiments or give new perspectives and comment on emerging approaches. On the contraty, our results show clearly that the largest part of preprint citations is found in the methodology section. This means that we can observe in such sections the construction of new scientific knowledge that is based on research that has not been validated by peers.

Our results also show that the lexical content of citation contexts differs significantly between preprint citations and citations to peer-reviewed articles. One possible explanation for this is that authors who cite preprints are aware of the nature of these works as non-validated knowledge and thus they use specific expressions and vocabulary to introduce these citations. More detailed analysis on the linguistic level is necessary to fully describe this phenomenon.

The motivations to cite preprints need to be further investigated. In fact, we have shown that preprints are most frequent in the Method section. These results can be put in resonance with the work of Small (2018), who classifies papers in the biomedical domain as method, type of method and non-method papers, based on the citation contexts in which they appear. Small (2018) establishes a relation between this classification and the perceived certainty or uncertainty of the underlying knowledge. In fact, the results show that methods and their outputs have higher certainty, while non-methods have higher uncertainty. Indeed, the concept of uncertainty is closely related to the nature of preprints as non-validated knowledge.

## Conclusion and perspectives

In this paper we study preprint citations in several different aspects: the progression of preprint citations over time, their relative frequencies in relation to the IMRaD structure of articles, their distributions over time, per preprint database and per PLOS journal. We have also compared the lexical content of preprint citation contexts with that of citation contexts of peer-reviewed publications. For our experiment, we have processed the PLOS corpus that covers 7 journals and a total of about 240,000 articles up to January 2021. From this corpus we have extracted 8460 preprint citations that were analysed in this paper.

We have shown that preprint citations have grown rapidly during the last years and that this growth is consistent with the creation and use of preprint repositories. Considering the IMRaD structure of articles, the Methods section, which contains a relatively small number of citations, stands out as carrying between 22 and 44% of all preprint citations. This particularity needs to be studied further in order to understand the reasons behind the fact that authors tend to place a relatively large number of preprint citations in the Methods section. We have observed the variations that exist between the different journals around these phenomena.

By performing a lexicometric analysis, we have shown that preprint citation contexts differ significantly from citation contexts of peer-reviewed publications. This means that authors make use of different lexical content when citing preprints compared to the rest of citations.

The main limitation of this study is the nature of the PLOS corpus. While the PLOS One journal is multidisciplinary and covers many fields, the rest of the journals are in several closely related disciplines such as Biology or Genetics. Therefore, a generalization of these results cannot be proposed. It would be necessary to extend this study to other scientific fields in order to obtain results about the phenomena that we have identified across different disciplines.

The next stage of this work will be to study of the contexts of citations to preprints in order to understand qualitatively the citation mechanisms behind citing preprints. In particular, a detailed analysis of the linguistic properties of preprint citation contexts in necessary to understand their functions in the rhetorical structure and argumentation of research, as well as their contribution to the construction of new knowledge.

## Data Availability

The Preprint Citations in PLOS dataset is available on Zenodo (Atanassova & Bertin, 2022).

## Footnotes

1 https://asapbio.org/preprint-servers, https://zenodo.org/record/4321522, https://en.wikipedia.org/wiki/List_of_academic_preprint_repositories, https://libguides.lib.hku.hk/preprint.
